# Feasibility of targeted early detection for melanoma: a population-based screening study

**DOI:** 10.1054/bjoc.2000.1183

**Published:** 2000-04-03

**Authors:** J Melia, C Harland, S Moss, J R Eiser, L Pendry

**Affiliations:** Cancer Screening Evaluation Unit, Section of Epidemiology, D Block, Cotswold Road, Sutton, Surrey SM2 5NG, UK; Department of Dermatology, Epsom and St Helier NHS Trust, Wrythe Lane, Carshalton, Surrey SM5 1AA, UK; Department of Psychology, University of Exeter, Washington Singer Laboratories, Perry Road, Exeter, EX4 4QG, UK

**Keywords:** melanoma, screening, early detection

## Abstract

The feasibility of targeted screening for cutaneous malignant melanoma in the UK using a postal questionnaire and invitation to screening by a consultant dermatologist was investigated in a population based cross-sectional survey. A total of 1600 people aged 25–69 years, stratified by the social deprivation score of wards within one general practice, were randomly selected from a population of 8000.1227 (77%) returned the questionnaire and 896 (56%) attended the screening clinic. Uptake was lower for men (*P*< 0.001), those aged under 50 (*P*< 0.001), people from deprived areas (*P*< 0.001) and skin types III and IV (men only, *P*< 0.001). Twenty per cent of women and 10% of men felt nervous about attending the clinic, but only 4% were worried by the questionnaire. The level of agreement between the self- and dermatologist's assessments of risk factors was best for hair colour (Kappa = 0.67, sensitivity 73% and specificity 98%). People tended to under-report their level of risk. Over 95% knew about at least one major sign, but 54% reported incorrect signs of melanoma. Targeted screening for melanoma in the UK will be hampered by difficulties in accurately identifying the target population. Strategies to improve skin self-awareness rather than screening should be developed and evaluated. © 2000 Cancer Research Campaign


British Journal of Cancer (2000) 82(9), 1605–1609
© 2000 Cancer Research Campaign
DOI: 10.1054/ bjoc.2000.1183, available online at http://www.idealibrary.com on IDEAL®

### Feasibility of targeted early detection for melanoma: a population-based screening study

**J Melia[1], C Harland[2], S Moss[1], JR Eiser[3] and L Pendry[3]**

[1]Cancer Screening Evaluation Unit, Section of Epidemiology, D Block, Cotswold Road, Sutton, Surrey SM2 5NG, UK; [2]Department of Dermatology, Epsom and St Helier NHS Trust, Wrythe Lane, Carshalton, Surrey SM5 1AA, UK; [3]University of Exeter, Department of Psychology, Washington Singer Laboratories, Perry Road, Exeter EX4 4QG, UK

**Summary** The feasibility of targeted screening for cutaneous malignant melanoma in the UK using a postal questionnaire and invitation to screening by a consultant dermatologist was investigated in a population based cross-sectional survey. A total of 1600 people aged 25–69 years, stratified by the social deprivation score of wards within one general practice, were randomly selected from a population of 8000.1227 (77%) returned the questionnaire and 896 (56%) attended the screening clinic. Uptake was lower for men ($P < 0.001$), those aged under 50 ($P < 0.001$), people from deprived areas ($P < 0.001$) and skin types III and IV (men only, $P < 0.001$). Twenty per cent of women and 10% of men felt nervous about attending the clinic, but only 4% were worried by the questionnaire. The level of agreement between the self- and dermatologist's assessments of risk factors was best for hair colour (Kappa = 0.67, sensitivity 73% and specificity 98%). People tended to under-report their level of risk. Over 95% knew about at least one major sign, but 54% reported incorrect signs of melanoma. Targeted screening for melanoma in the UK will be hampered by difficulties in accurately identifying the target population. Strategies to improve skin self-awareness rather than screening should be developed and evaluated. © 2000 Cancer Research Campaign

**Keywords**: melanoma; screening; early detection

In the UK, high profile campaigns to promote the early detection of melanoma in the general public (MacKie and Hole, 1992; Melia, 1995) led to increased detection of thin melanomas. However, the effects of the campaigns on mortality have been inconclusive, and have generated a large workload. Given the low prevalence of melanoma in the UK, targeted screening of those with a high risk of melanoma may be a cost-effective strategy (Elwood, 1994; Little et al, 1995; Tornberg et al, 1996; Jackson et al, 1998). The feasibility of identifying a high-risk group has been investigated in two studies conducted in the UK. Little et al (1995), reporting on families registered with an affluent general practice in Wessex, found that the public's self-assessment of mole counts was poor except for moles on the front of the trunk which is not necessarily the site associated with a high risk of melanoma (Farinas-Alvarez et al, 1999). Jackson et al (1998) reported a good reliability for assessing risk factors, but this study was based on attenders to general practice in Cheshire who volunteered to attend for screening (388 out of 3105 patients).

In this paper, results are reported from the first population-based screening study of a random sample of 1766 adults aged 25–69 years by a consultant dermatologist. The main aim was to investigate the feasibility of screening in a broader mix of social class groups than the previous studies (Little et al, 1995; Jackson et al, 1998). Results are reported on the accuracy with which people assessed their risk factors of melanoma, the prevalence of risk factors, the findings of a total skin examination, and anxiety associated with the study. Data on attitudes towards screening are reported in a separate paper (Eiser et al, submitted for publication).

*Received 11 November 1999*
*Revised 21 January 2000*
*Accepted 21 January 2000*

*Correspondence to:* J Melia

### MATERIALS AND METHODS

The intervention consisted of a postal questionnaire survey asking questions about risk factors for melanoma and attitudes towards screening. A letter from the general practitioner (GP) and dermatologist was enclosed which offered an appointment for screening for skin cancer by a consultant dermatologist (CH) at the local district hospital. Ethical approval was obtained from the Merton, Sutton and Wandsworth Health Authority.

The study population came from one general practice in Carshalton, Surrey with a population of 8000. A random sample of households with people aged 25–69 years were stratified by the social deprivation score of wards (Carstairs and Morris, 1992) within the practice catchment area. The sample size was adequate to study a prevalence of 10% (95% confidence intervals of ± 2.5%) for risk factors, and a sensitivity of 75% ± 10% to identify by self-assessment a population at high risk of melanoma.

Data collection took 1 year from November 1997. Up to three mailings of the questionnaire and letter were sent. Non-attenders to the screening clinic were asked about the reasons for non-attendance. The questionnaire asked about information on risk factors for melanoma (Appendix) (MacKie, 1989), demographic details and concerns about the invitation for screening. The question on skin type asked about six groups: White skin which never tans and always burns (I), White skin which burns at first and tans with difficulty (II), White skin which tans easily and burns rarely (III), White or olive skin which never burns and always tans (IV), Brown skin (V), Black skin (VI) (MacKie, 1989).

Data on atypical moles and a history of sunburn, both risk factors for melanoma (Bataille et al, 1998), were not collected because of uncertainties about accuracy of reporting by the general public (Little et al, 1995). A skin check guide described the type of moles to be counted, and how to assess freckling.

**Table 1** Distribution (%) of demographic factors in responders and non-responders

| Characteristics | Non-responders (Q–C–) | Responders: Questionnaire (Q+C–) | Questionnaire and clinic (Q+C+) | Total | Number | Significance |
|---|---|---|---|---|---|---|
| Sex | | | | | | |
| Males | 28 | 21 | 51 | 100 | 751 | *P* < 0.001 |
| Females | 19 | 20 | 61 | 100 | 845 | |
| Total | 23 | 21 | 56 | 100 | 1596 | |
| Number | 369 | 331 | 896 | | | |
| Age | | | | | | |
| <50 years | 27 | 22 | 51 | 100 | 1019 | *P* < 0.05 |
| ≥50 years | 16 | 19 | 65 | 100 | 577 | |
| Total | 23 | 21 | 56 | 100 | 1596 | |
| Number | 369 | 331 | 896 | | | |
| Carstairs | | | | | | |
| 1st quintile (affluent) | 16 | 15 | 69 | 100 | 282 | |
| 2nd quintile | 21 | 23 | 56 | 100 | 438 | |
| 3rd quintile | 24 | 16 | 60 | 100 | 404 | |
| 4th quintile | 23 | 27 | 50 | 100 | 209 | *P* < 0.001 |
| 5th quintile (deprived) | 33 | 25 | 42 | 100 | 263 | |
| Total | 369 | 331 | 896 | | 1596 | |

A mole was defined as ‘a skin mark which is usually dark brown but can be black or light brown. It appears before the age of 35 years and does not appear to change colour in the sun. Moles can be flat (not raised above the skin surface) or raised (felt above the skin surface)’. Only moles ≥ 2mm or more in diameter were counted. A freckle was defined as ‘a light brown mark with an irregular edge that appears or goes darker in the sun, and is usually less than 2 mm in diameter.’

Moles and freckling were illustrated in photographs and diagrams. People were asked about their knowledge of the early signs of melanoma based on a recommended checklist (MacKie, 1990). Demographic details included date of birth, sex, age when left full-time education and living with a partner or spouse. Participants were asked whether they were pleased or concerned to receive the questionnaire, and to be invited to the screening clinic.

At the clinic, held 1 day per week from 14:30 to 18:30 h, the dermatologist asked about skin type, natural hair colour, a personal history of skin cancer and a family history of melanoma. A whole body examination was conducted excluding areas covered by underwear. Data on moles and freckling were collected using the same definitions as for the questionnaire. Anyone with a possible skin cancer was asked to see their GP so that they could be referred formally for excision/biopsy.

Descriptive statistics were used to study the uptake rate, and prevalence of risk factors in relation to various demographic factors in STATA. The accuracy with which people self-report risk factors for melanoma was assessed using measures of **sensitivity** (the proportion of people reporting a risk factor out of all those reported to have the risk factor by the dermatologist), **specificity** (the proportion of people who do not report a risk factor out of all those reported not to have the risk factor by the dermatologist), and **Kappa** statistics (the level of agreement between the individual and dermatologist taking into account agreement occurring by chance). The values of Kappa range from 0 (no agreement) to 1 (total agreement) and within this range, values < 0.4 are considered to represent poor agreement, 0.4–0.74 fair to good agreement and ≥ 0.75 excellent agreement (Landis and Koch, 1997). Logistic regression was used to study the relation of demographic factors to uptake rates, and to agreement of self-assessed risk factors to the dermatologist’s observations.

Knowledge of the early signs of melanoma was studied in a multiple choice question containing nine possible correct statements interspersed with three incorrect statements. A score of correct answers ranging from 0 to 12 was created by scoring 1 for each of the nine correct signs that were ticked and scoring 1 for each of the three incorrect signs that were not ticked.

### RESULTS

A total of 1766 people were randomly selected from the GP list. Of these, 164 were found to have moved or left the practice, and two had died. Out of 1600, 1277 (77%) returned the questionnaire and, of these, 896 (56% of 1600) also attended the clinic. Compared with those who had only returned the questionnaire (Q+C–), and those who did neither (Q–C–), those who had both returned the questionnaire and attended the clinic (Q+C+) had higher proportions of women than men ($P < 0.001$), those aged 50 or more than younger adults ($P < 0.001$) and people from the least deprived than more deprived areas ($P < 0.001$, Table 1). The most frequent reasons for non-attendance to the clinic were being too busy (47%), believing they had no risk of skin cancer (27%), and inconvenience of clinic time (18%). Only 1.5% objected to study. Thirty-eight people of Black or Brown ethnic group were excluded from subsequent analyses.

Comparing Q+C+ with Q+C– in regression analyses, men were more likely to attend the clinic if they were aged 50 or more (odds ratio (OR) 1.04, 95% confidence interval (CI) 1.02–1.06), or had skin type I or II (OR 1.71, 95% CI 1.26–2.32). Women were less likely to attend if they came from deprived areas (OR 0.8, 95% CI 0.70–0.93).

Only 4% found the questionnaire distressing. The proportion of people who were nervous about the thought of coming for a skin check was low (Q+C+ 15% and Q+C– 17%), but it was twice as

*© 2000 Cancer Research Campaign*

**Table 2** Distribution (%) of phenotypic factors compared between questionnaire and clinic data

| Questionnaire data | Clinic data | | | |
|---|---|---|---|---|
| Hair colour[a] | Black/brown | Blond | Red | Total no. |
| Black/brown | 85 | 18 | 15 | 620 |
| Blond | 13 | 80 | 12 | 172 |
| Red | 2 | 2 | 73 | 46 |
| Total | 100 | 100 | 100 | |
| Number | 700 | 97 | 41 | 838 |
| Reporting red hair | | | | |
| | Sensitivity | Specificity | Kappa 0.67 | |
| | 73% | 98% | | |
| Skin type | III, IV | II | I | Total no. |
| III, IV | 97.1 | 45.6 | 4.4 | 459 |
| II | 2.5 | 54.4 | 71.7 | 378 |
| I | 0.4 | 0.0 | 23.9 | 39 |
| Total | 100 | 100 | 100 | |
| Number | 243 | 474 | 159 | 876 |
| Reporting of skin type I | Sensitivity 24% | Specificity 100% | Kappa 0.34 | |
| Self-report of skin types I or II compared with clinic report of skin type I | Sensitivity 96% | Specificity 63% | Kappa 0.36 | |
| Freckles | None/few | Moderate | Many | Total no. |
| None/few | 80.7 | 62.4 | 29.9 | 498 |
| Moderate | 18.4 | 35.5 | 52.7 | 307 |
| Many | 0.9 | 2.1 | 17.4 | 65 |
| Total | 100 | 100 | 100 | |
| Number | 347 | 189 | 334 | 870 |
| Reporting many freckles | Sensitivity 17% | Specificity 99% | Kappa 0.19 | |
| Self-report of moderate or many freckles compared with clinic report of many freckles | Sensitivity 70%<br>70% | Specificity 74%<br>74% | Kappa 0.13 | |

[a] 32 people reporting grey/white hair were excluded as the dermatologist concentrated on recording their natural hair colour at age 20.

high in women than men (20% and 10% respectively, $P < 0.001$). Twice as many in Q+C+ as in Q+C– wanted to find out more about their personal risk of skin cancer (74% vs 35%, $P < 0.001$).

The levels of agreement between self-reports of risk factors and those recorded by the dermatologist were poor to fair for skin type, freckling and mole counts (Tables 2 and 3). The level of agreement was highest for hair colour when the reporting of red hair was compared with other hair colours as one group (Kappa 0.67) (Table 2). Counting moles on the head and neck was more accurate among those with none or few freckles (Kappa 0.32) compared with those having moderate or many freckles (Kappa 0.17). The prevalence of freckling, and level of agreement for this factor or for mole counts did not vary with time of year.

There was a tendency for people to report the highest risk category less frequently than the dermatologist. Overall, 4% self-reported skin type I, 7% many freckles and 7% many moles. A high sensitivity could be achieved, although at a loss to specificity, if the self-reports of the two highest risk categories for each factor were compared with the dermatologist's assessment of the highest risk category (Tables 2 and 3). For example, for skin type, sensitivity increased from 24% to 96%, and specificity decreased from 100% to 63%, by comparing those self-reporting skin types I and II with those recorded as skin type I by the dermatologist. However, the prevalence of this potential target group for screening was high (48%).

In regression analyses restricted to those reported by the dermatologist to have skin type I (number in analysis 158), self-reports was more likely to agree with this assessment for males than females (OR 3.1, 95% CI 1.4–6.8) after allowing for the effects of age, social deprivation, age left education and living with a partner. For those with many freckles and those with many moles, the agreement was unrelated to demographic factors.

Ninety-three per cent knew about 'growing', 41% 'having different colours' and 35% 'having a ragged outline' as signs of melanoma. Seventy-six per cent also knew about 'changing shape'. Poor discriminatory signs 'bleeding, oozing or crusting', and 'a change in sensation such as itching or pain' were reported by 74% and 69% respectively. Fifty-four per cent reported one or more incorrect signs: scaly, hairy or raised above the skin surface as signs of melanoma. In regression analyses to study the relation between a score of correct answers (range 0–12) and demographic factors, significantly lower scores were found in men compared with women (regression coefficient –0.72, 95% CI –1.03 to –0.42) and in those who had left education by 16 (regression coefficient –0.22, 95% CI –0.37 to –0.07).

Eighteen people with a suspicion of skin cancer were advised to see their GPs for biopsy under the normal pattern of care. Twelve basal cell carcinomas and two melanomas were histologically confirmed. Both melanomas were thin (Breslow thickness < 0.76 mm) and were found on the backs of men aged more than 50 years.

### DISCUSSION

Cost-effective screening requires a good uptake rate, a practical and accurate method for identifying the target population, and a choice of a target population that ensures a high yield of melanomas with consideration of both economic and psychological costs.

© 2000 Cancer Research Campaign

**Table 3** Prevalence, level of agreement (kappa), sensitivity and specificity comparing questionnaire and clinic data for mole counts comparing one category for each variable against the rest

| Variable | Questionnaire | Clinic | Kappa | Sensitivity | Specificity |
|---|---|---|---|---|---|
| Moles on head and neck ≥10 | | | | | |
| Prevalence | 4% | 6% | 0.22 | 23% | 97% |
| Total number | 37/867 | 48/867 | | | |
| Moles on trunk ≥10 | | | | | |
| Prevalence | 27% | 33% | 0.35 | 50% | 84% |
| Total number | 237/870 | 288/870 | | | |
| Moles on arms and hands ≥10 | | | | | |
| Prevalence | 23% | 43% | 0.34 | 42% | 91% |
| Total number | 200/870 | 371/870 | | | |
| Moles on legs and feet ≥10 | | | | | |
| Prevalence | 16% | 29% | 0.40 | 42% | 94% |
| Total number | 143/869 | 250/869 | | | |
| Overall quantity of moles | | | | | |
| Many | | | | | |
| Prevalence | 7% | 19% | 0.32 | 26% | 98% |
| Total Number | 60/869 | 168/869 | | | |
| Moderate/many in questionnaire vs many in clinic data | | | | | |
| Prevalence | 37% | 19% | –0.1 | 74% | 72% |
| Total number | 320/869 | 168/869 | | | |

This study has shown that overall a good response (77%) can be achieved from a postal questionnaire but this varies according to demographic factors. The uptake to a screening clinic was lower (56%), but this could be improved by focusing on an older age group, among whom the incidence of melanoma is higher, offering a wider choice of clinic times and providing health education to increase awareness about skin cancer, particularly in more deprived areas (Eiser et al, submitted for publication). In this paper, 54% of people reported incorrect signs for melanoma.

The response rates to the questionnaire and uptake of screening were lower than that in a study conducted in an affluent general practice population in Wessex (84% and 89% respectively) (Little et al, 1995). The social mix, wider choice of appointment times, and use of the GP surgery may account for the higher response in Wessex.

The accuracy with which risk factors for melanoma could be identified by a postal questionnaire was disappointing, and was lower than in Wessex or Cheshire. One of the most important risk factors for melanoma is having a large number of naevi but there was a general reluctance for people to put themselves in the extreme group having a large number of moles. One option for targeted screening might be to select people who report moderate to high risk, to ensure that all high risk people are included in an intervention. However, this would increase the proportion of people falling into the target group (e.g. 4% and 42% self-reporting skin types I and II respectively).

The postal questionnaire raised anxiety about skin cancer in only a small proportion of cases (< 4%). Although about 15% of people said that they were nervous about attending for a skin check this did not seem to be related to attendance rates. It was not feasible to study anxiety over a longer period. The long-term psychological effects of screening would be important in future research testing new strategies.

The detection rate of melanomas (113 per 100 000) was very high compared with the incidence rate in 25- to 69-year-olds in England and Wales (8.9 per 100 000). Another study offering screening by a dermatologist in the workplace showed a similar high detection rate (Curley et al, 1993). Self-selection bias and the high detection expected in the prevalence round of screening may explain this finding.

The options for improving the early detection of melanoma include general population professional screening, targeted screening of a high risk group, self-screening and skin awareness. General population screening would not be cost-effective in the UK because of the low incidence. Targeted screening by GPs has been proposed (Little et al, 1995; Jackson et al, 1998) but this may benefit only a small proportion of melanomas and has considerable implications for workload, and psychological and economic costs (Keeley, 1995; Sinclair, 1998). Advice on skin awareness could be provided to encourage regular self-skin checks through a practice nurse (Ringborg et al, 1991). This advice should aim to improve awareness of the early signs of melanoma, demonstrated to be lacking in this survey. Recognition of melanoma by the general public might be improved by using a chart with photographs representing a range of benign, borderline and malignant lesions. The effectiveness of this approach would need to be evaluated in terms of self-referral rates to GPs, and sensitivity and specificity of detecting melanomas.

The results of previous feasibility studies for targeted screening of melanoma in the UK may have been too optimistic. Future strategies to improve and maintain both a high level of early detection by the general public and accurate recognition of suspicious lesions by GPs need to be evaluated in terms of workload, psychological outcome and economic costs.

### ACKNOWLEDGEMENTS

We thank the Cancer Research Campaign and the Department of Health for funding the study. We are grateful to Mrs Christine Ellis and Dr Derek Coleman for co-ordinating the project and preparation of the datafile, to Mrs Sue Ritson and Mrs Theresa Beire for organizing the skin check clinic and follow-up of non-responders, and to Dr Colin Greaves for advice on questionnaire design.

 *© 2000 Cancer Research Campaign*

**REFERENCES**

Bataille V, Grulich AE, Sasieni P, Swerdlow A, Newton Bishop JA, McCarthy WH, Hersey P and Cuzick J (1998) The association between naevi and melanoma in populations with different levels of sun exposure: a joint case–control study of melanoma in the UK and Australia. *Br J Cancer* **77**: 505–510

Carstairs V and Morris R (1992) *Deprivation and Health in Scotland*. Aberdeen University Press: Aberdeen

Curley RK, Taylor FG, Marsden RA, Cox J and McLaughlin CA (1993) Screening for skin cancer: experience of an occupational health screening programme. *Occup Med Oxf* **43**: 207–210

Eiser JR, Pendry L, Greaves CJ, Melia J, Harland CC and Moss SM. Attitudes towards early detection and personal risk of cutaneous malignant melanoma in a UK population. (submitted for publication).

Elwood JM (1994) Screening for melanoma and options for its evaluation. *J Med Screen* **1**: 22–38

Farinas-Alvarez C, Rodenas JM, Herranz MT and Delgado-Rodriguez M (1999) The naevus count on the arms as a predictor of the number of melanocytic naevi on the whole body. *Br J Dermatol* **140**: 457–462

Jackson A, Wilkinson C, Ranger M, Pill R and August P (1998) Can primary prevention or selective screening for melanoma be more precisely targeted through general practice? A prospective study to validate a self administered risk score. *Br Med J* **316**: 34–39

Keeley D (1995) Screening for melanoma risk is misguided [comment]. *Br Med J* **310**: 916–916

Landis JR and Koch GG (1977) The measurement of observer agreement for categorical data. *Biometrics* **33**: 159–174

Little P, Keefe M and White J (1995) Self screening for risk of melanoma: validity of self mole counting by patients in a single general practice. *Br Med J* **310**: 912–916

MacKie RM (1989) *Malignant Melanoma. A Guide to Early Diagnosis*. Edinburgh

MacKie RM (1990) Clinical recognition of early invasive malignant melanoma. *Br Med J* **301**: 1005–1006

MacKie RM, Freudenberger T and Aitchison TC (1989) Personal risk-factor chart for cutaneous melanoma. *Lancet* **2**: 487–490

MacKie RM and Hole D (1992) Audit of public education campaign to encourage earlier detection of malignant melanoma. *Br Med J* **304**: 1012–1015

Melia J (1995) Early detection of cutaneous malignant melanoma in Britain. *Int J Epidemiol* **24**: S39–44

Ringborg U, Lagerlof B, Broberg M, Mansson Brahme E, Platz A and Thorn M (1991) Early detection and prevention of cutaneous malignant melanoma: emphasis on Swedish activities. *Med Oncol Tumor Pharmacother* **8**: 183–187

Sinclair R (1998) Commentary: Start with the KISS principle. *Br Med J* **316**: 38–39

Tornberg S, Mansson-Brahme E, Linden D, Ringborg U, Krakau I, Karnell J, Landegren J, Brandberg Y and Hakulinen T (1996) Screening for cutaneous malignant melanoma:a feasibility study. *J Med Screen* **3**: 211–215

### APPENDIX

**What type of skin do you have?**

| | | |
|---|---|---|
| Black skin | | 1 |
| Brown skin | | 2 |
| White or olive skin which never burns and always tans | (IV) | 3 |
| White skin which tans easily and burns rarely | (III) | 4 |
| White skin which burns at first and tans with difficulty | (II) | 5 |
| White skin which never tans and always burns | (I) | 6 |

If you have BLACK OR BROWN SKIN, you have a very low risk of skin cancer and you do not need to answer the questions on pages 1–5. However, it would be helpful if you could answer the questions on the last page of the questionnaire.

**What is your natural hair colour?**

| | |
|---|---|
| Black/dark or light brown | 1 |
| Fair/blond | 2 |
| Red/ginger | 3 |
| Other | 4 |

**To what extent do you have freckles?**

| | |
|---|---|
| No freckles | 1 |
| Few freckles only on one or two parts of body | 2 |
| Moderate freckling | 3 |
| Many large freckles usually greater than 2 mm on face, forearm and upper back | 4 |

**How many moles, 2 mm diameter or greater, have you on your skin?**

| | | | | | | | | | | | | |
|---|---|---|---|---|---|---|---|---|---|---|---|---|
| Head & neck | None | 1 | 2 | 3 | 4 | 5 | 6 | 7 | 8 | 9 | 10 | or more |
| Trunk | None | 1 | 2 | 3 | 4 | 5 | 6 | 7 | 8 | 9 | 10 | or more |
| Arms & hands | None | 1 | 2 | 3 | 4 | 5 | 6 | 7 | 8 | 9 | 10 | or more |
| Legs & feet | None | 1 | 2 | 3 | 4 | 5 | 6 | 7 | 8 | 9 | 10 | or more |

**Overall, how moley do you think you are?**

| | |
|---|---|
| | 0 |
| Few moles | 1 |
| Moderate number of moles | 2 |
| Many moles | 3 |

**Have you had any relatives with melanoma?** (usually a dark/black form of skin cancer)

| | |
|---|---|
| Yes | 1 |
| No | 2 |
| Don’t know | 3 |

**If YES:** which of your relatives had melanoma?

| | |
|---|---|
| Mother or father | 1 |
| Blood-related aunt or uncle | 2 |
| Brother or sister | 3 |
| Son or daughter | 4 |
| Other relative | 5 |

**Have you had a melanoma previously?**

| | |
|---|---|
| Yes | 1 |
| No | 2 |
| Don’t know | 3 |

© 2000 Cancer Research Campaign

## References

[PDF_00376] Bataille V., Grulich A., Sasieni P., Swerdlow A., Newton Bishop J., McCarthy W., Hersey P., Cuzick J. (1998). The association between naevi and melanoma in populations with different levels of sun exposure: a joint case-control study of melanoma in the UK and Australia.. Br J Cancer.

[PDF_00388] Elwood J. M. (1994). Screening for melanoma and options for its evaluation [see comment].. J Med Screen.

[PDF_00390] Fariñas-Alvarez C., Ródenas J. M., Herranz M. T., Delgado-Rodríguez M. (1999). The naevus count on the arms as a predictor of the number of melanocytic naevi on the whole body.. Br J Dermatol.

[PDF_00393] Jackson A., Wilkinson C., Ranger M., Pill R., August P. (1998). Can primary prevention or selective screening for melanoma be more precisely targeted through general practice? A prospective study to validate a self administered risk score.. BMJ.

[PDF_00397] Keeley D. (1995). Screening for melanoma risk is misguided.. BMJ.

[PDF_00399] Landis J. R., Koch G. G. (1977). The measurement of observer agreement for categorical data.. Biometrics.

[PDF_00401] Little P., Keefe M., White J. (1995). Self screening for risk of melanoma: validity of self mole counting by patients in a single general practice.. BMJ.

[PDF_00404] MacKie R. M. (1990). Clinical recognition of early invasive malignant melanoma.. BMJ.

[PDF_00407] MacKie R. M., Freudenberger T., Aitchison T. C. (1989). Personal risk-factor chart for cutaneous melanoma.. Lancet.

[PDF_00409] MacKie R. M., Hole D. (1992). Audit of public education campaign to encourage earlier detection of malignant melanoma.. BMJ.

[PDF_00411] Melia J. (1995). Early detection of cutaneous malignant melanoma in Britain.. Int J Epidemiol.

[PDF_00413] Ringborg U., Lagerlöf B., Broberg M., Månsson-Brahme E., Platz A., Thörn M. (1991). Early detection and prevention of cutaneous malignant melanoma: emphasis on Swedish activities.. Med Oncol Tumor Pharmacother.

[PDF_00417] Törnberg S., Månsson-Brahme E., Lindén D., Ringborg U., Krakau I., Kärnell R., Landegren J., Brandberg Y., Hakulinen T. (1996). Screening for cutaneous malignant melanoma: a feasibility study.. J Med Screen.

